# Industrial Robot Control System with a Predictive Maintenance Module Using IIoT Technology

**DOI:** 10.3390/s25041154

**Published:** 2025-02-13

**Authors:** Andrzej Wojtulewicz, Patryk Chaber

**Affiliations:** Institute of Control and Computation Engineering, Faculty of Electronics and Information Technology, Warsaw University of Technology, ul. Nowowiejska 15/19, 00-665 Warsaw, Poland; patryk.chaber@pw.edu.pl

**Keywords:** industrial robot, diagnostics, prediction, IIoT, CNC, HP HMI, MQTT, communication protocols

## Abstract

The article describes solutions in the field of diagnostics of a control system based on a CNC and the cooperation with an industrial robot. The industrial robot is controlled directly from the CNC. Data exchange between the CNC and the robot controller allows for collecting the most important process data from the robot. Then, calculations are performed in the PLC using a number of functions to obtain consumption indicators of individual robot components. The data were visualized on the HMI screens of the CNC. Additionally, a dedicated interface was prepared to share these data using the MQTT protocol for IIoT solutions. The entire solution was implemented and then deployed in a real station. The presented solution is an extension of the possibilities of operating an industrial robot by CNC towards diagnostics and early failure prevention.

## 1. Introduction

The work presented in the article concerns the diagnostics of consumption of individual parts of an industrial robot controlled by a CNC (Computerized Numerical Control). In current industrial revolution era, there is a visible trend to invest in advanced systems for continuous diagnostics using this type of equipment. Special measuring systems are used in combination with analytical software. Advanced algorithms are used to process measurement data to predict possible failures. This allows for planning maintenance and interventions in advance, instead of reacting to problems that already occur. Thanks to the early detection of potential problems, the maintenance department can plan maintenance and repairs in an organized and efficient manner, minimizing downtime. This planning can be integrated with other management systems, allowing for resource management. Based on the analysis, the system generates alarms for the maintenance department. Alarms can inform us about the need to carry out inspection, maintenance, or repair. In the long term, the collected data and conclusions from the predictive diagnostics system can be used to optimize production processes and improve machine design. These systems also enable a better understanding of the causes of failures and contribute to the continuous improvement of operational processes. The article presents implementations of solutions based on the PLC (Programmable Logic Controller) and the analysis of data from the robot. In addition, the prepared interface allows for data collection using IIoT (Industrial Internet of Thing) technology for superior IT (Information Technology) systems.

The following part of the chapter presents examples from the literature concerning the topic of the article. The second chapter presents the general structure of the system as well as the most important elements used in the implementation of the work. The IIoT technology used to provide diagnostic data is described. Then, the implementations of the most important diagnostic functions implemented in the PLC calculated on the basis of process data from the robot are described. The third chapter presents the implemented visualization screens with sample data collected from the actual implementation of the robot in the station. The last chapter summarizes the most important results obtained during the work.

### 1.1. Related Work

The aim of Ref. [1] is to present a practical implementation of a network architecture capable of collecting data from industrial robots, in order to later implement “One-Class Novelty Detection” models—identification of data that significantly differ from patterns observed during machine learning. The architecture uses only well-known automation components, such as a PLC. The implementation was performed on a real assembly line in the automotive industry, where it was possible to demonstrate the effectiveness of detecting anomalies in the work of robots.

Ref. [2] describes the process of several possible solutions for predictive diagnostics of robots from data collection, through the creation of an appropriate data set, to the use of machine learning algorithms. The algorithms were evaluated using appropriate performance metrics in terms of accuracy and precision, which were proven to be effective in predicting robot damage. Additionally, the impact of the Principal Component Analysis (PCA) technique on the obtained results, used to reduce the dimensionality of the data, was checked.

Ref. [3] presents a methodology for creating a data acquisition strategy for predictive diagnostics of industrial robots. The paper describes the components of six-axis robots that are susceptible to failure in the automotive industry, such as power supplies, servos, or gears, and it also presents data sources that are a source for detecting faults of robot components. The most commonly used parameters for fault detection are motor currents and vibration analysis.

Ref. [4] presents the application of an LSTM recurrent neural network for predictive diagnostics of industrial robots. The model predicts the day of failure based on alarm analysis, achieving high prediction accuracy. The significant help this solution provides to the maintenance department in effective maintenance planning is described.

Ref. [5] developed a predictive diagnostic model for industrial robots based on an artificial neural network, using MTTF values and system failure history. Data were acquired using an ERP system, and the results showed the effectiveness of predicting failures by the MLP structure, minimizing costs related to unplanned downtime. Reliability analysis and spare part forecasts based on the Poisson distribution allowed for the creation of an optimal spare part list, ensuring production efficiency is at 85%.

In Ref. [6], an approach based on the analysis of robot currents is proposed to detect accuracy errors of individual robot joints for predictive diagnostics. The methodology is based on the analysis of the time series of currents to predict deviations in robot accuracy. The results confirmed a strong correlation between teh selected three-phase current properties and robot accuracy. The developed predictive model showed high efficiency and good fit.

In Ref. [7], a predictive maintenance method for semiconductor wafer transport robots was developed using the K-means clustering algorithm to analyze data from acceleration sensors attached to the robot arm. The data were subjected to normalization and denoising processes, and the data analysis enabled the prediction of the appropriate maintenance moment. Simulations showed that the method allows for real-time prediction, minimizing the risk of sudden failures.

The aim of Ref. [8] was to propose a robotic cell reliability optimization method based on the digital twin (DT). A machine learning model was used to detect and classify faults of critical components and predict their remaining service life (RUL). Reliability analysis using RBD and FTA methods showed that parallel connections of critical components minimize the risk of failure of the entire system.

Ref. [9] concerns the implementation of predictive diagnostics for industrial robots, only using data from existing sensors, without the need to install new ones. Torque characteristics were used to predict robot anomalies. Different algorithms were tested and the results showed that the hybrid approach (Exponentron + GPR) is the best. It was also shown that low data quality significantly worsens the results, so it is necessary to have high-quality data and a good sampling rate.

Ref. [10] concerns predictive fault detection in robots using motor current signal analysis (MCSA), aimed at monitoring and assessing the condition of the robot drive system. The applied method allows for fault detection in the drive system. The novelty is in mastering the classical limitations of signal analysis in the frequency domain, extending the range beyond the steady state. Signals outside the steady states showed rich information content.

Ref. [11] presents reliability engineering methods that can be used to predict the failure probability of mobile robots. A new modification of the mean time to failure concept is proposed, taking into account the influence of operating conditions on the failure rate. Furthermore, these techniques are applied to the design of mobile robot tasks, enabling the prediction of the probability of task completion.

### 1.2. Article Contribution

The article presents the implementation of a diagnostic system for an industrial robot controlled directly from the CNC [12]. The robot performs loading tasks [13] of a workpiece for automatic machining [14]. In order to ensure the highest production reliability [15], diagnostic solutions [16] were presented, allowing for the earlier response of maintenance services with regard to servicing and ensuring the continuity of the entire system [17]. Based on the data collected from the industrial robot [18], the consumption rates of individual parts are calculated, thanks to which it is possible to prevent system failures, thus reducing the costs related to the unplanned downtime of the station. Dedicated HMI (Human Machine Interface) screens have been designed on the CNC, which allow for insight into current data from the industrial robot. Maintenance services can use these data to plan the replacement of a specific assembly or perform service activities related to lubrication in advance. Implementations in the PLC and the visualization of HMI panels have been presented. To increase functionality and adapt to the new standards of Industry 4.0 [19], the system has been supplemented with the ability to download data via MQTT (Message Queue Telemetry Transport) [20,21] for the needs of IIoT solutions [22].

The most important features of the presented solution are as follows:

- Extending the scope of access to the diagnostic data of the CNC system, in particular, through the MQTT broker [23] for IIoT technology [24].
- Expanding the robot control mechanism with diagnostic functions and predicting the servicing of robot parts [25].
- The implementation made in the PLC, which eliminates the need for additional devices. Possible implementation on analogous control systems [26].
- The visualization of all diagnostic information directly on the CNC. Additionally, making these data available through IIoT [27].
- The possibility of collecting other process data regarding machining on the CNC machine.
- The functions for prediction are open and can be freely modified and further developed for robots from other manufacturers.
- The system is open to the possibility of communication with robots from other manufacturers, e.g., FANUC.
- The developed solution can be extended for use with any devices (sensors or controllers) supporting the SLMP. It is also possible to easily modify the implementation with an additional MODBUS TCP protocol using Ethernet [28].

## 2. Materials and Methods

The article presents the most interesting fragments of programs implemented in the PLC calculating consumption. The implementation was made in the ST (Structured Text) language. From the point of view of PLC programming, this is a dedicated solution for performing calculations.

**Figure 1:**
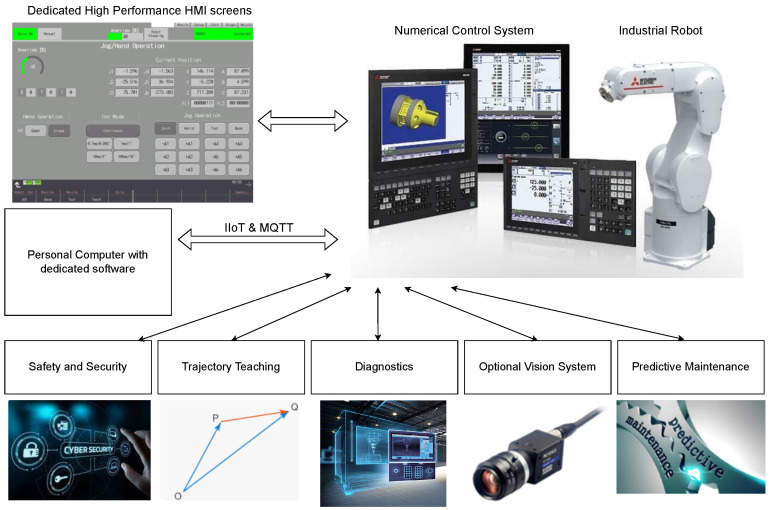
Control system structure.

The structure of the system is shown in Figure 1. The main CNC system M80V Mitsubishi Electric performs the task of controlling the lathe in the first channel. The second channel is dedicated to controlling the Mitsubishi Electric RV2F industrial robot. The CR800D robot controller is connected to the CNC system via Ethernet, thanks to which it is possible to send all the necessary process data (CC-Link IE Field Basic) from the robot as well as its control (Direct Robot Control). For the convenient setting of the robot’s operating parameters and for performing its diagnostics, dedicated screens displayed directly on the CNC have been prepared. NC Designer 2 software version 2.1.9 was used to implement the visualization. Access to the CNC screens is possible using VNC (Virtual Network Computing). To obtain IIoT functionality, a dedicated PC was used, which has an MQTT broker providing data from the field of industrial robot diagnostics. The broker is supplied with data using the SLMP, which retrieves data directly from the CNC’s PLC. As an alternative to a PC, it is possible to use a microcontroller with analogous functions, which has extended the scope of the access to diagnostic data of the CNC. In addition to the implementation of diagnostic functions, the robot’s learning and trajectory playback functions have also been implemented. The operator’s safety is ensured by a dedicated safety system for which software for redundant PLCs has been developed. It is also possible to use an optional vision system for the inspection of the manufactured part.

### 2.1. IIoT Interface

MQTT is a communication protocol designed for resource-constrained devices in Industrial Internet of Things (IIoT) solutions. It was designed for use with small data sizes and high-latency flows. It has low requirements, and it is reliable and efficient. Thanks to this, it has become a basic element in IIoT solutions. It can be used in IIoT devices that run on batteries, sending data to collecting and processing servers. This solution is ideal for devices that require low power consumption and minimal data transfer makes it suitable for IIoT applications. The principle of operation of the MQTT protocol is based on the principle of publishing and subscribing messages. The device publishing data sends them to a specific topic on the MQTT broker, which acts as a center for managing the flow of messages. Other devices can subscribe to specific topics and receive any data sent to them. An important aspect of MQTT is its implementation efficiency—the small size of the communication frame base and its high compression allow for fast and reliable communication between devices.

**Figure 2:**
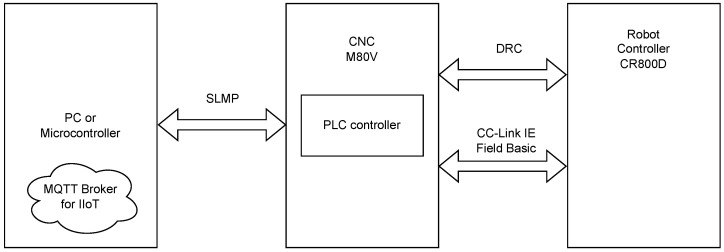
Dataflow for IIoT.

The dataflow of data used in the presented solution is shown in Figure 2. Sending important information about current process values from the robot controller to the PLC was done using CC-Link IEF Basic communication. Furthermore, some data were sent using the DRC (Direct Robot Control) protocol used, in general, to control the connected robot. All of the data were displayed on dedicated screens, and some of them were taken to calculating diagnostic coefficients.

The SLMP (Seamless Message Protocol) is a communication protocol used by Mitsubishi Electric. It was developed to facilitate communication between industrial automation devices. The CC-Link IEF Basic protocol used to exchange data between the PLC and the robot controller is based on the SLMP. It is characterized by fast and reliable communication. It works in client-server mode, the client (PC or microcontroller) sends a request to the server, and the server (PLC) processes the request and sends a response. The main functionality of the SLMP connection used in this application was reading data, i.e., reading specific areas of the PLC memory. For the CNC, it is necesssary to configure parameter #1489 – SLMP_ON to 1, activating the SLMP server on the PLC.

Robot data that were sent to the PLC or data that were calculated in the PLC were sent to the PC or microcontroller, which acted as a protocol converter. The communication was done with the PLC via the SLMP, reading the necessary data, and then preparing them using the MQTT communication protocol. In this way, the data were prepared for master devices using IIoT technology. The solution does not include security mechanisms against cyberattacks. The communication protocols and Ethernet connections were used in their standard versions and access protection was left to external network devices and the network administrator.

### 2.2. Grease Consumption

**Figure 3:**
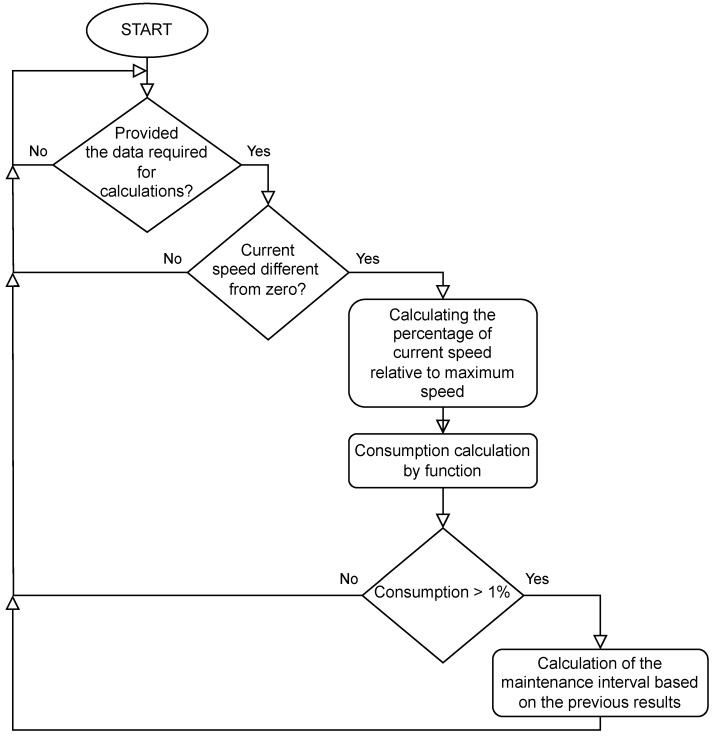
Grease consumption function flow diagram.

Grease consumption is calculated based on the speed of the motor in a given joint. The time interval specified in the settings can become longer or shorter. Consumption is not calculated if the motors are not running. The function block presented in Listing 1 is responsible for calculating the consumption of grease. The following calculations are carried out according to the block diagram shown in Figure 3.

**Listing 1.** Robot consumption grease function block.

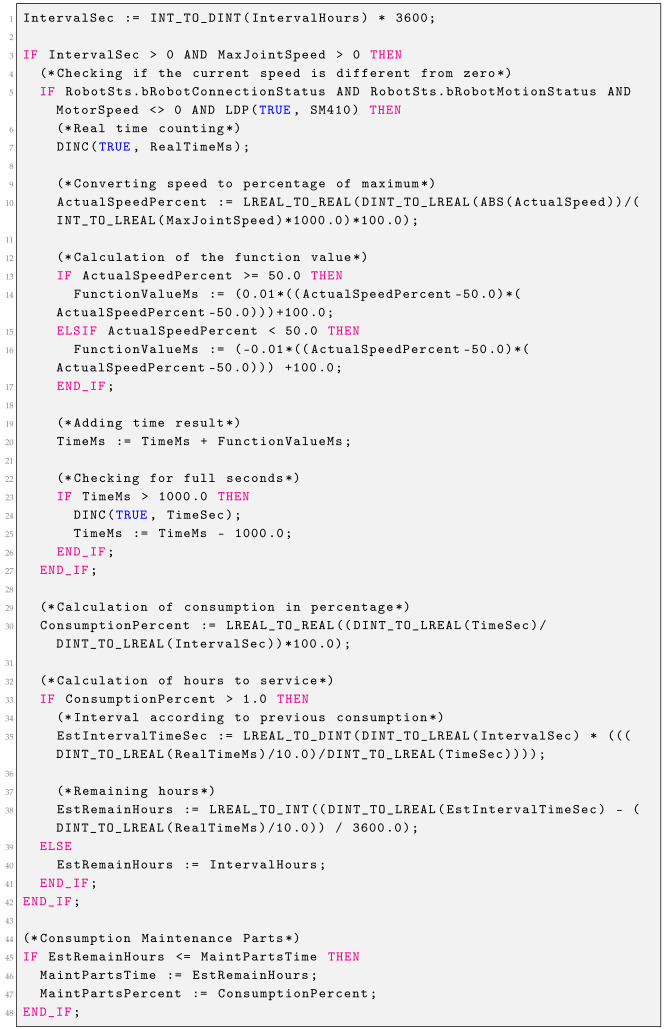



If the current speed of the motor is different from zero, the variable RealTimeMs is incremented every 100 ms, which counts the time the lubricant is running without any penalty. Then, the percentage of the current motor speed relative to the maximum speed is calculated. This value in percentage is passed as an argument to the quadratic functions. The function for an argument above 50% returns values greater than 100 ms, while for an argument below 50%, it returns values less than 100 ms. This means that operation at speeds above 50% is penalized, while at speeds below 50% is rewarded, extending the grease replacement interval. Grease consumption is calculated based on the time generated using quadratic functions. By comparing this time with the real grease operation time, it is possible to estimate the grease replacement interval based on the consumption to date and calculate the remaining hours to be serviced with such robot operations.

### 2.3. Timing Belt Consumption

**Figure 4:**
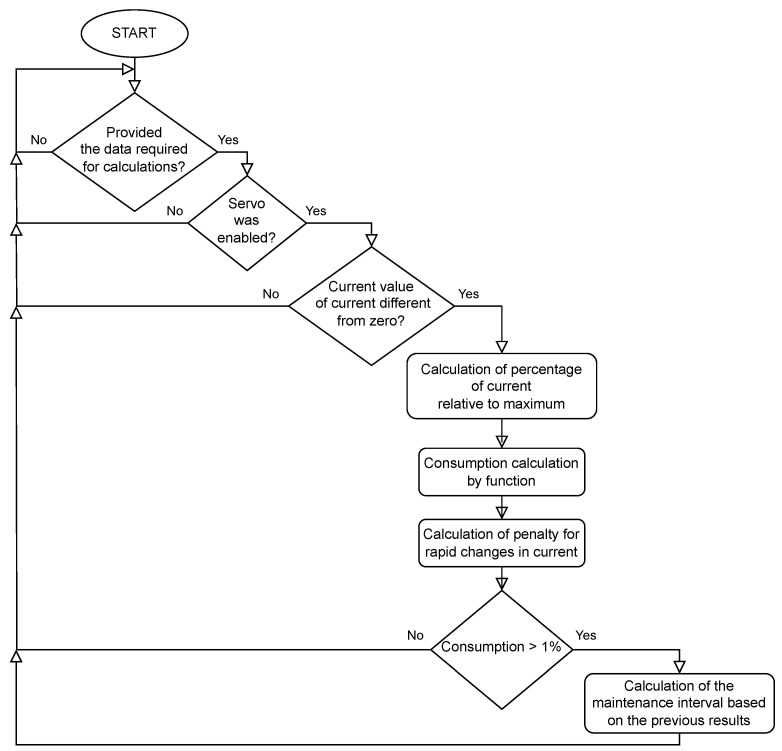
Timing belt consumption function flow diagram.

Timing Belt consumption is calculated based on the value of the currents and the rate of change. The time interval specified in the settings may not become longer, but it may become shorter. Using the robot at high speeds and with sudden changes in direction will result in a shorter interval. Consumption is calculated from the moment the Servo On is turned on, if the currents are different from zero—consumption can also be calculated while the robot is stationary. The function block presented in Listing 2 is responsible for calculating the consumption of the timing belt. The following calculations are carried out according to the block diagram shown in Figure 4.

If the current is different from zero, the variable RealTimeMs is incremented every 100 ms, which counts the belt running time without any penalty. Then, the percentage of the current value relative to the maximum current value is calculated. This value in percentage is passed as an argument to a quadratic function. The function for an argument above 20% returns values greater than 100 ms, while for an argument below 20%, it returns a value of 100 ms. This means that consumption increases when 20% of the current is exceeded. In addition, the value of the change in current as a percentage of the maximum current is calculated. If the value exceeds 5%, then, additional ms are added using a linear function. The values from both functions are summed. Belt consumption is calculated based on the time generated by the functions. By comparing this time with the real time of belt operation, it is possible to estimate the replacement interval on the basis of the consumption to date and calculate the remaining hours to be serviced with such robot operations.

**Listing 2.** Timing belt consumption function block.

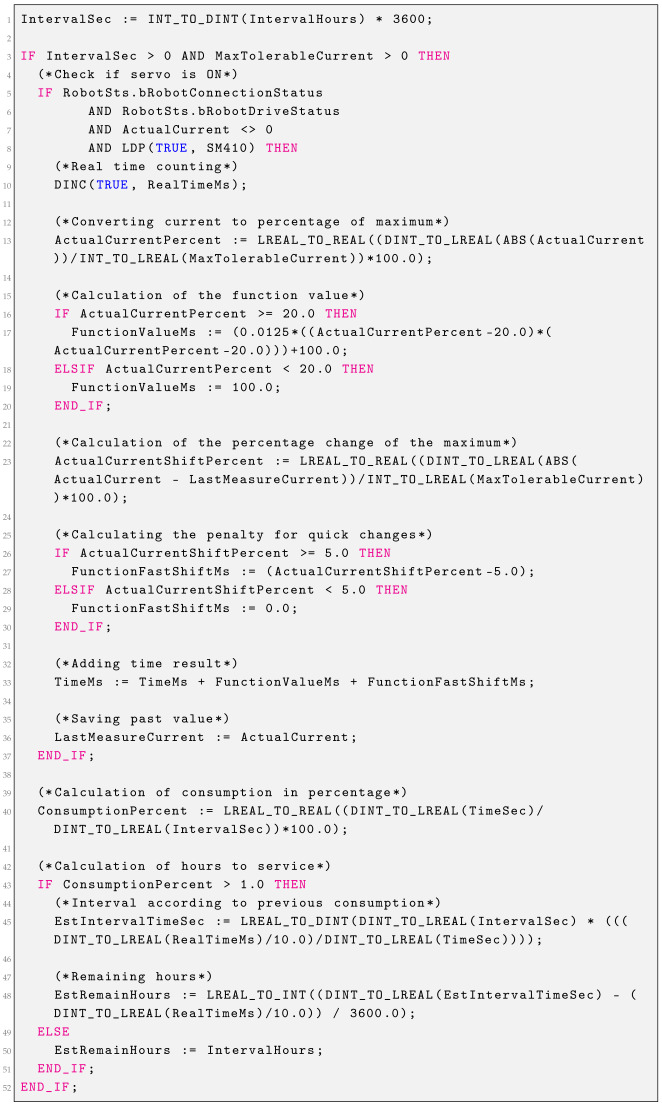



### 2.4. Gear Consumption

Gear consumption is calculated based on the current values, the rate of their change, and the motor speed. The time interval specified in the settings cannot be extended, but it may be shortened. Using the robot at high speeds and with sudden changes in direction will result in a shortened interval. Consumption is counted from the moment Servo On is turned on, if the currents are different from zero—consumption can also be counted when the robot is stationary.

If the current is different from zero, the RealTimeMs variable is incremented every 100ms, which counts the gear operation time without any penalties. Then, the percentage of the current current value relative to the maximum is calculated. This value of the percentage is passed as an argument of the quadratic function. For an argument above 20%, the function returns values greater than 100 ms, while for an argument below 20%, it returns the value 100 ms. This means that the consumption increases after exceeding 20% of the current. In addition, the value of the current change is calculated as a percentage of the maximum current. If this value exceeds 5%, then, additional ms are added using a linear function. Then, the percentage of the current engine speed relative to the maximum is calculated. This percentage value is passed as an argument of the quadratic function. For an argument above 30%, the function returns values greater than 0 ms, while for an argument below 30%, it returns the value 0 ms. The values from the three functions are added together. The consumption of the gear unit is calculated based on the time generated by the functions. Comparing this time with the real working time of the gear unit allows us to estimate the replacement interval, based on the current consumption, and calculate the remaining hours for inspection for such robot operations.

### 2.5. Power on Time, Servo on Time

The function presented in Listing 3 is used to calculate the working time of the robot and the working time of the drives of individual joints. It is possible to set the allowable number of hours of operation of a given element. After counting the set number of hours, an appropriate message will be appear on the alarm screen, suggesting the performance of the appropriate service actions.

**Listing 3.** Power on time.

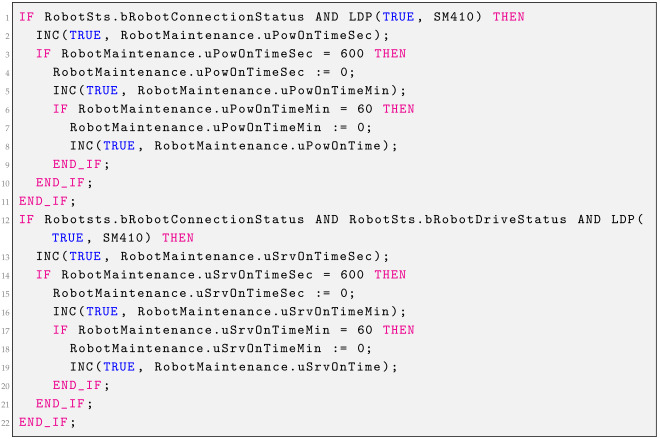



### 2.6. Bearing Consumption

Bearing consumption is calculated based on the values of currents and motor speeds. The time interval specified in the settings cannot be extended, but it may be shortened. Using the robot at high speeds and with sudden changes in direction will result in a shorter interval. Consumption is counted from the moment Servo On is turned on if the currents are different from zero—consumption can also be counted when the robot is stationary. The function block presented in Listing 4 is responsible for calculating the consumption of bearing.

If the present current is different from zero, the RealTimeMs variable is incremented every 100ms, which counts the operating time of the bearing without any penalties. Then, the percentage of the current current value relative to the maximum is calculated. This percentage value is passed as an argument of the quadratic function. For an argument above 20%, the function returns values greater than 100 ms, while for an argument below 20%, it returns the value 100 ms. This means that the consumption increases after exceeding 20% of the current.

Then, the percentage of the current motor speed relative to the maximum is calculated. This percentage value is passed as an argument of the quadratic function. For an argument above 30%, the function returns values greater than 0 ms, while for an argument below 30%, it returns 0 ms. The values of both functions are added together.

The bearing consumption is calculated based on the time generated by the functions. Comparing this time with the actual operating time of the bearing allows us to estimate the replacement interval, based on the current consumption, and to calculate the remaining hours until inspection for such robot operations.

**Listing 4.** Consumption bearing function block.

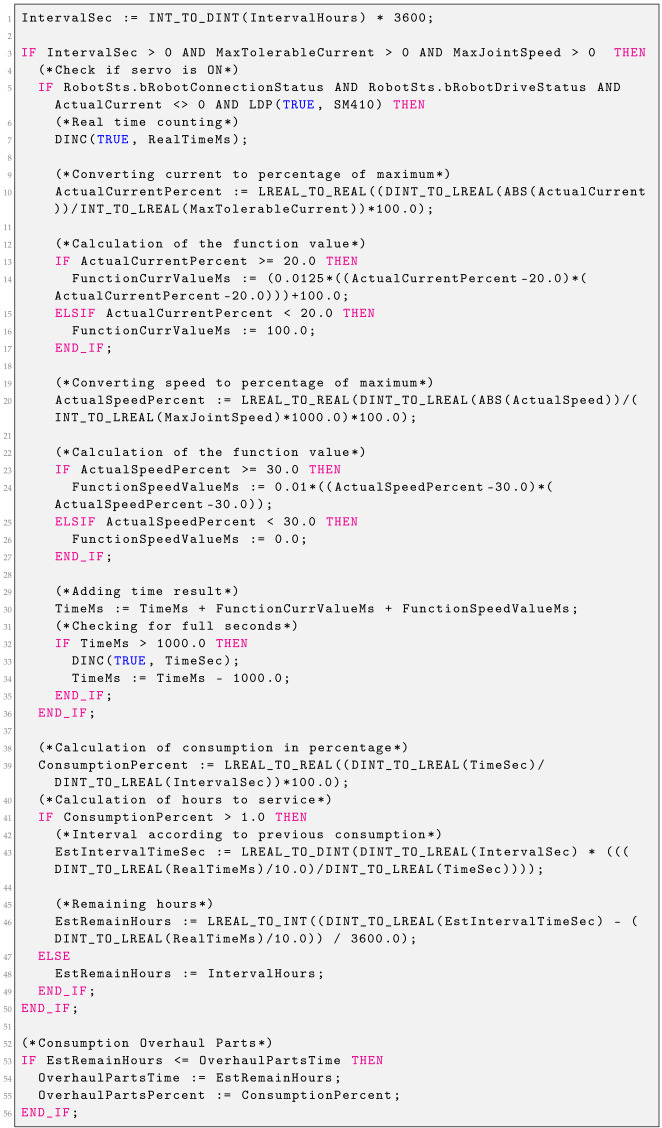



### 2.7. Gear Prediction

**Figure 5:**
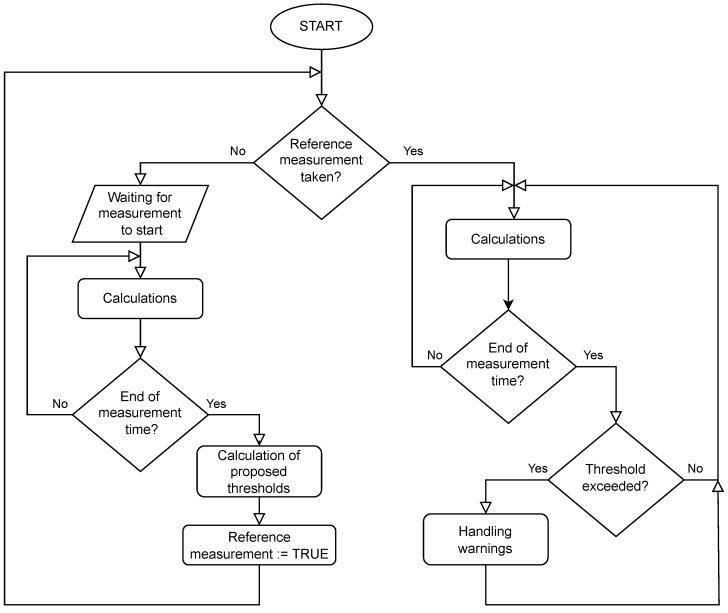
Gear prediction function flow diagram.

Calculations for detecting gear malfunctions are performed according to the flow diagram presented in Figure 5.

At first, a reference measurement is performed, in which the following are calculated:

- Average of positive current values.
- Average of negative current values.
- Maximum current.
- Minimum current.
- Average positive change in current value.
- Average negative change in current value.
- Maximum positive change in current value.
- Maximum negative change in current value.
- (Average load).
- (Maximum load).

After the reference measurement is completed, the variables that inform us about long-term and short-term malfunctions are calculated. The following are added up for long-term malfunctions: average of positive current values, average of negative current values, average positive change in current value, and average negative change in current value. On the other hand, the following are added up for short-term malfunctions: maximum current, minimum current, maximum positive change in current value, and maximum negative change in current value. Then, the proposed thresholds are calculated. If the reference measurement has been performed, measurements are taken to examine the operation of the gear.

Later measurements are the same as the reference measurement, i.e., calculating certain values for a specified period of time, summing them up appropriately, and comparing them with thresholds. If any of them is exceeded, an appropriate warning is produced.

## 3. Results

### 3.1. Description of Robot Screens

The dedicated screens for operating and monitoring the robot have several common elements. These include a bottom menu and a top panel displaying the most important current statuses of the robot.

The Maintenance menu consists of the following sections:

- Total—overall consumption and tear of the robot.
- MainteParts (maintenance parts)—consumption of the first group of parts.
- OvrhaulParts (overhaul parts)—consumption of the second group of parts.
- AbnormlDetect (abnormalities detection)—detection of abnormalities.
  − Gear J1–J3.
  − Gear J4–J6.
- Log—history of services performed.
  − MaintParts (maintenance parts).
  − OvrhaulParts (overhaul parts).
  − Other.
- OprInf (operating info)—information on operating times.
- Setting—parameter settings.
- Warning—warning threshold settings.
- Reset—deleting alarms and performing services.

### 3.2. Total

**Figure 6:**
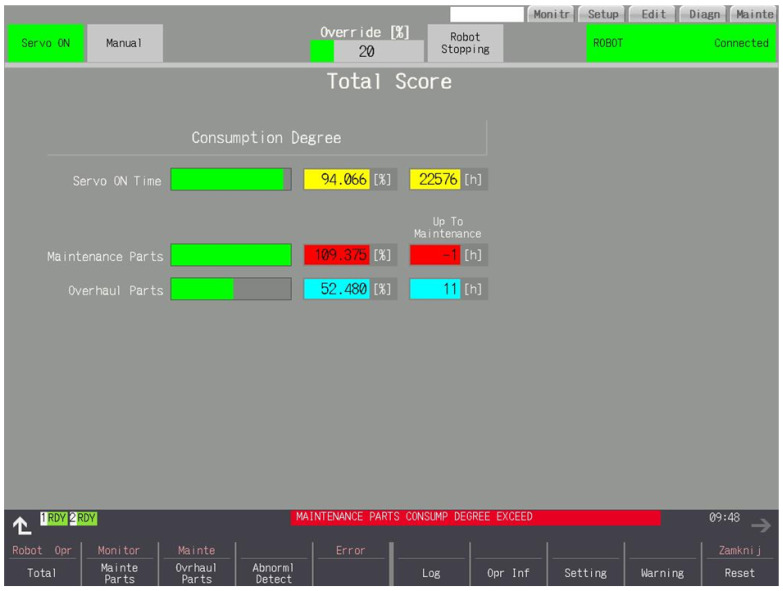
Number of hours Servo On exceeded the warning threshold. Maintenance parts exceeded 100%—alarm triggered. Overhaul parts are worn to a degree that does not cause an alarm condition.

The number of hours worked with Servo On and the use of the Servo On interval in percentages are shown graphically and numerically in Figure 6. Consumption consists of both part groups. In both cases, the consumption of the part from the group with the lowest time to service is displayed.

### 3.3. Maintenance Parts

**Figure 7:**
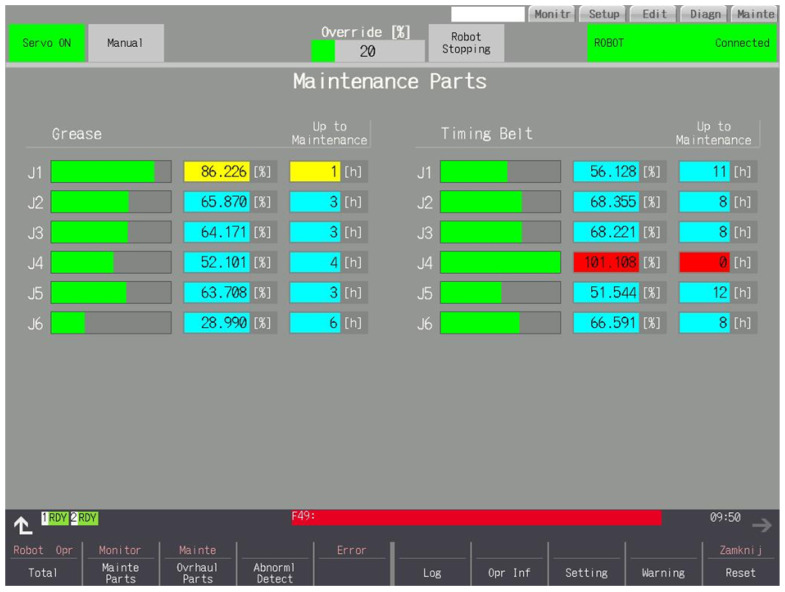
Consumption of belts and lubricants.

The lubricant and belt consumption are shown in graphical and numerical form in Figure 7. Lubricant consumption is calculated based on the speed of the motors. The interval can become longer or shorter. Consumption is not counted if the motors are not running. Belt consumption is calculated based on the value of currents and the rate of change of the current values. The interval cannot be extended. Using the robot at high speeds and with sudden changes in direction will result in a shorter interval. Consumption is counted from the moment the Servo On is turned on, if the currents are different from zero—consumption can also be counted while the robot is stationary. The estimated time to service is displayed if the consumption exceeds 1%. This is calculated based on the robot’s work to date.

### 3.4. Overhaul Parts

**Figure 8:**
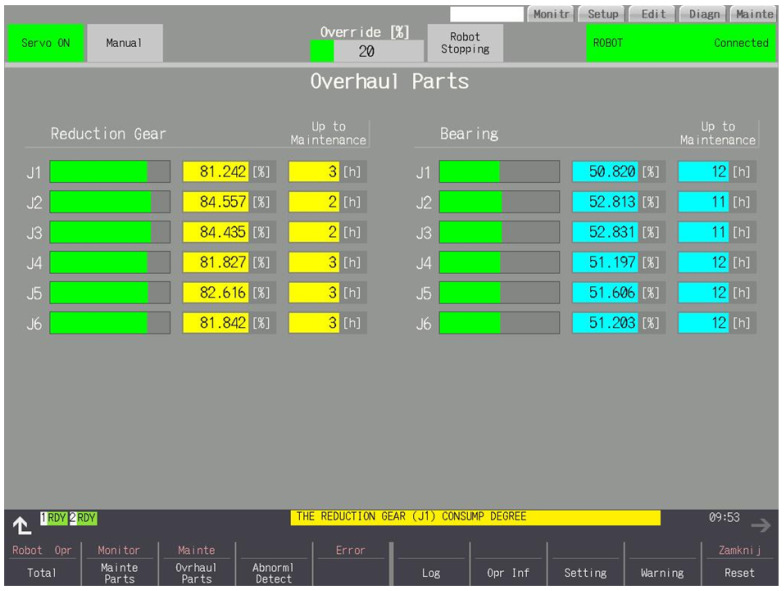
Consumption of gear and bearing.

Gearbox and bearing consumption in graphical and numerical form in Figure 8. Gear consumption is calculated on the basis of current values, the rate of change of the current values and the speed of motors. The interval cannot be extended. Using the robot at high speeds and with sudden changes in direction will result in a shorter interval. Consumption is counted from the moment the Servo On is turned on, if the currents are different from zero—consumption can also be counted while the robot is stationary. Bearing consumption is calculated based on the currents and speed of the motors. The interval cannot be extended. Using the robot at high speeds and heavy loads will result in a shorter interval. Consumption is counted from the moment the Servo On is turned on, if the currents are different from zero—consumption can also be counted while the robot is stationary. The estimated time to service is displayed if the consumption exceeds 1%. This is calculated based on the robot’s work to date.

### 3.5. Abnormality Detection

**Figure 9:**
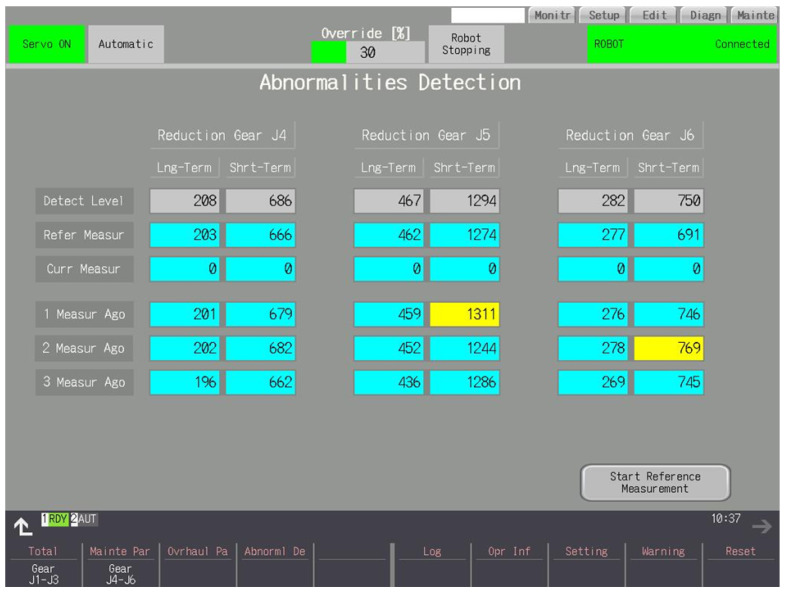
J4–J6 joints. The reference run took place. This follows several measurements analyzing the robot’s operation.

This section has two screens that display the results for detecting gear malfunctions. A reference measurement is required for operation—the longer the better. If one has not been taken, the message “No Reference Measurement” will appear on a red background at the bottom of the screen shown in Figure 9. This can be done by starting the robot in automatic mode and pressing the “Start Reference Measurement” button, which will change the caption to “During Reference Measurement” during the reference run. When it is completed, the reference result values from the measurement and the suggested detection levels will appear. After a reference run, the abnormality detection function is immediately activated. The robot’s operation is analyzed, and the results are analyzed at certain time intervals. If the results exceed the abnormality detection level, a warning is reported, which is made cleared with the physical Reset button. Abnormalities are divided into those that have been detected for most of the examined time, “Long-term”, and temporary, “Short-term”, disturbances in the gear system. Detection levels can be modified by the user. The results are stored and displayed from the last three detection periods. If any of them exceeds the detection level, its background turns yellow. Results from the current sample are displayed, for example, one minute after the start of the period. The values of the long-term results can increase as well as decrease during the ongoing analysis. Short-term results, on the other hand, do not decrease during the test sample. The result is calculated based on the average and maximum value of currents and the rate and magnitude of current changes. The results vary depending on the work performed by the robot. So, the threshold should be set for the current working conditions of the robot.

### 3.6. LOG

**Figure 10:**
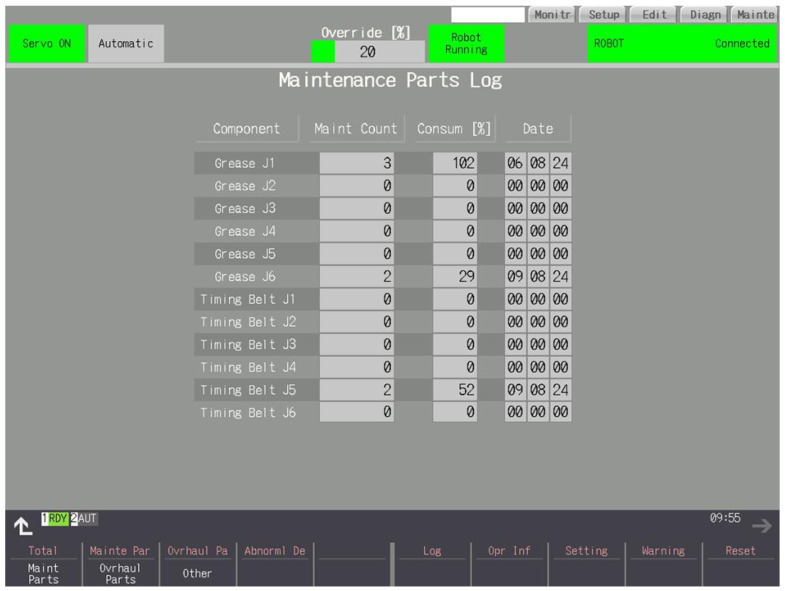
The first of the log screens—belts and greases.

The screen presented in Figure 10 shows how many services have been performed on a particular part, at what consumption, and when the last replacement/overhaul was performed.

### 3.7. Operating Info

**Figure 11:**
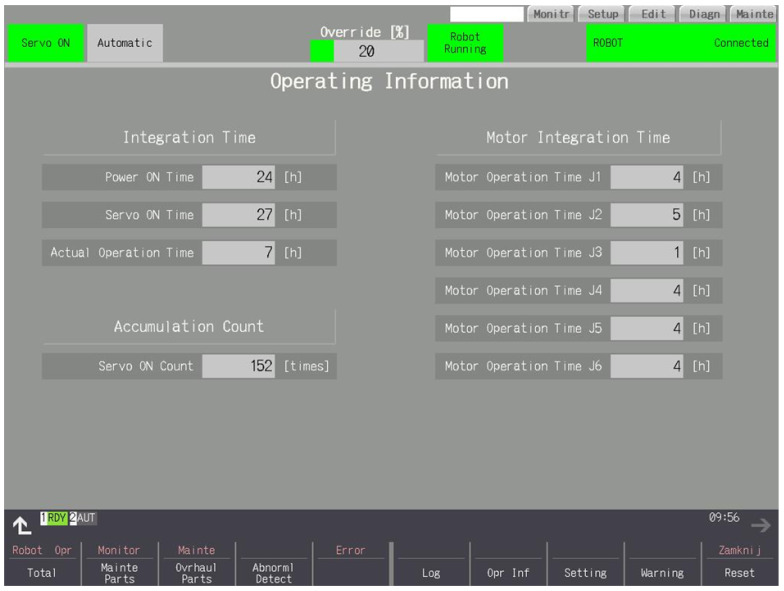
Total times since last full inspection.

Figure 11 shows the times since the last full inspection of the robot controller was turned on, the time since the servo was engaged, and the time during which the robot moved. In addition, it shows the number of servo engagements. The right side of the screen displays the total motor times of each joint.

### 3.8. Settings

**Figure 12:**
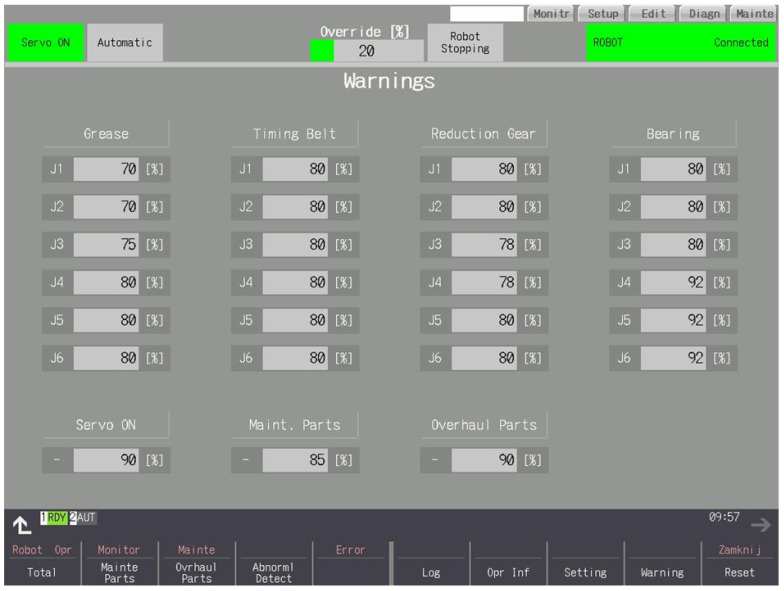
Warning levels.

**Figure 13:**
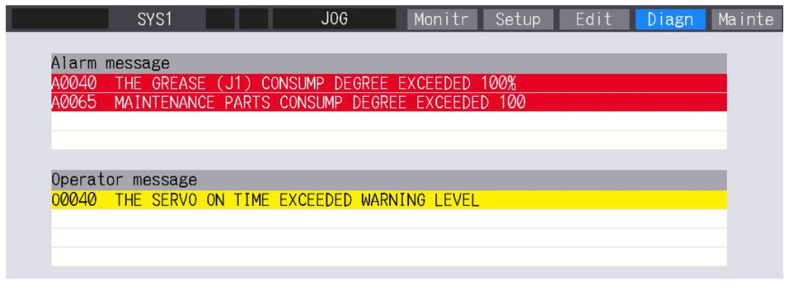
Warning samples.

Configurable consumption levels of given parts at which a warning is to be reported (10%–99%)—presented in Figure 12. The warning is a yellow message with the exact contents and the numerical value of the consumption and the remaining hours to be serviced for a given part highlighted in yellow. The warning appears when the CNC panel is turned on again—presented in Figure 13.

### 3.9. Reset

**Figure 14:**
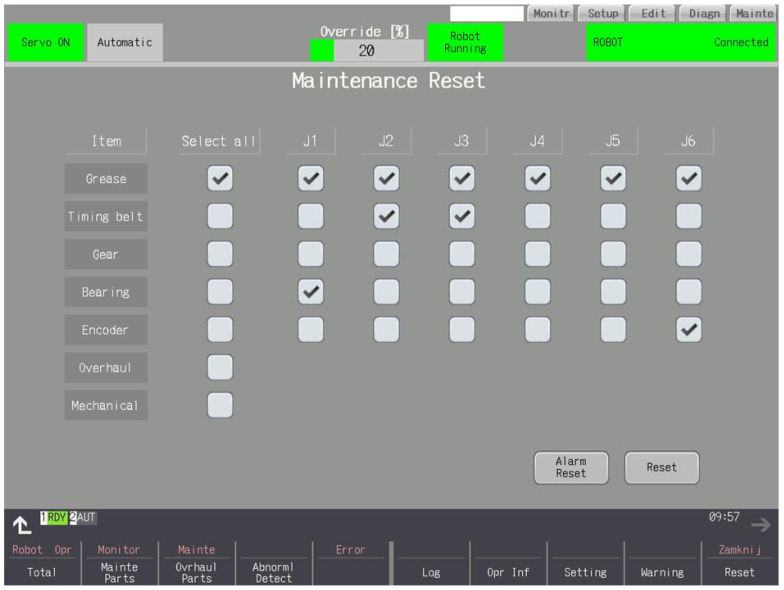
Clearing alarms and recording inspections performed.

A screen for clearing alarms and logging the completed inspections is presented in Figure 14. Alarms are represented by a red message with the exact contents and with red highlighting the numerical value of the consumption and the remaining hours to overhaul a given part after exceeding 100% consumption. Resetting the alarm only removes the message. The alarm appears when the CNC panel is turned on again. The reset records the date and current consumption of the part, which is displayed on the log screen.

- Grease—reset of grease consumption.
- Timing belt—reset of belt consumption.
- Reduction gear—reset of gear consumption.
- Bearing—reset of bearing consumption.
- Encoder—reset of motor timing.
- Overhaul, mechanical—reset of all consumption and times.

## 4. Conclusions

An original implementation of an industrial robot control system was presented. The solution was based on the DRC mechanism and multi-channel synchronization in the CNC. Dedicated programs were developed in the PLC for diagnostics based on numerical data from the industrial robot. In combination with the HMI screens, they allow for quick and efficient diagnostics of the robot during the production of parts on a lathe operated by the aforementioned industrial robot. Detecting potential failures before they occur allows for planning maintenance without disrupting the production process. This prevents expensive failures and unplanned downtime, which translate into savings for the end customer. Regular maintenance based on the actual condition of the robot extends its life and work efficiency. The collected data are analyzed in the PLC. The results are presented on the HMI screen of the CNC and also sent to the computer, where they can be made available to superior systems thanks to IIoT technology. The solution was tested on a real industrial station, where the robot picked up parts from the magazine, fed them to the lathe, where the machining was then carried out in accordance with the specified technology.

The presented solutions were prepared for Mitsubishi Electric robots controlled by DRC via a CNC. It is possible to extend the scope of application of the solution for FANUC robots also controlled by DRC via a Mitsubishi CNC. In the case of other robot manufacturers, it is possible to use the same mechanisms while ensuring data delivery using alternative data protocols supported by the considered industrial robots. The extension of functionality can also be implemented within the mechanisms of data collection on the PC side and their further analysis in order to detect additional anomalies in the work of the station.

## Data Availability

Upon request from the authors.
